# Severe Malaria Not Responsive to Artemisinin Derivatives in Man Returning from Angola to Vietnam

**DOI:** 10.3201/eid2107.141448

**Published:** 2015-07

**Authors:** Pascal Ringwald, Arjen M. Dondorp

**Affiliations:** World Health Organization, Geneva Switzerland (P. Ringwald);; Faculty of Tropical Medicine, Mahidol University, Bangkok, Thailand (A.M. Dondorp)

**Keywords:** Plasmodium falciparum, malaria, parasites, artimisinin, artesunate, dihydroartemisinin clindamycin, piperaquine, quinine, doxycycline, resistance, Western Cambodia, Africa, Angola, Greater Mekong Subregion, Vietnam

**To the Editor:** Partial artemisinin-resistant *Plasmodium falciparum* malaria, characterized by delayed parasite clearance after treatment with artesunate or artemisinin-based combination therapy, was first detected in western Cambodia and has now spread to or emerged de novo in 5 countries of the Greater Mekong Subregion (GMS) (*1*). However, most reported cases of malaria have been in Africa, and detecting artemisinin and multidrug resistance in Africa will have consequences for policy and containment plans (*2*). 

Thus, vigilant monitoring is pivotal, and it is therefore with great interest that we read the case report on a patient in Vietnam with severe *P.* *falciparum* malaria, acquired in Angola in 2013, that was not responsive to artesunate or several other antimalarial combinations (*3*). We believe that there are several issues that challenge the conclusion that artemisinin resistance has reached Angola: 1) the phenotypic and genotypic characteristics of the infecting strain in this patient were very different from artemisinin-resistant strains in the GMS; 2) pharmacokinetic issues cannot be ruled out; and 3) perhaps of most relevance, the study documents severely delayed clearance of multiple strains in this polyclonal *P. falciparum* infection, suggesting splenic hypofunction as an important contributor.

The parasite clearance half-life calculated with the World Health Organization (WHO) online slope analyzer from the log linear segment of the clearance curve after start of artesunate therapy was 102.5 hours, which is ≈10 times longer than observed in the most artemisinin-resistant parasites in Cambodia. Postpublication genotyping of the infecting strain provided by the authors to WHO showed a wild-type Kelch (K13) gene, which is a recently discovered molecular marker for artemisinin resistance strongly correlated to the resistant phenotype in the GMS (*1*).

No pharmacokinetic assessment was made, and subtherapeutic artesunate and dihydroartemisinin (as well as clindamycin, piperaquine, quinine, and doxycycline) blood concentrations cannot be excluded. The intravenous artesunate regimen used differed from the WHO guideline of 2.4 mg/kg on admission, after 12 h, then daily. Pharmacokinetic modeling of the split doses used in the described case indicate that this dosing schedule results in <20% artesunate and dihydroartemisinin blood concentrations. In addition, quality issues in the artesunate batch might have played a major role. Batch no. 511002 used for this patient (not 511004 as mentioned in the article) was manufactured by Pharbaco (Hanoi, Vietnam) in April 2011 and had a shelf-life of 3 years; it was quality controlled and passed the quantitative testing by high pressure liquid chromatography in January 2014 (National Institute of Drug Quality Control, Vietnam). However, according to information shared with WHO, a test for clarity after reconstitution was not performed, whereas other samples from the same batch had failed this specific test, which led the Drug Administration of Vietnam to withdraw this batch from the market. The patient was subsequently treated with nasogastric-administered dihydroartemisinin/piperaquine and quinine plus doxycycline. Reduced intestinal absorption in this severely ill patient, related to reduced splanchnic blood flow, could have resulted in reduced bioavailability (*4*).

Host factors can affect parasite clearance. In this case, the parasitological response to artesunate and clindamycin, dihydroartemisinin/piperaquine, quinine, and doxycycline were all unusually slow. Functional asplenia results in very slow parasite clearance after artesunate treatment, resembling the clearance characteristics in the described case (*5*). This interpretation is supported by finding genotypes representing >2 clones of parasites persisting >1 week after treatment with multiple antimalarial drugs. It seems very unlikely that this patient harbored multiple highly artemisinin-resistant parasite strains. Dead circulating intraerythrocytic parasites in patients who have hyposplenia can be recognized morphologically, but the article does not provide details on this.

Circulation of multidrug resistant malarial strains in sub-Saharan Africa can have disastrous consequences, and it is critical to detect its arrival at an early stage. The case report by Van Hong et al. implies the unlikely event of independent emergence of multidrug resistant strains in a traveler from Vietnam in Angola, without evidence of local declining artemisinin-based combination therapy efficacy. WHO and partners are investigating the phenotype and genotype of parasite strains from the same geographic area in Angola to address the concerns raised above. We believe that this single case report is insufficient to raise the alarm.
